# *In silico* evaluation of the role of lisdexamfetamine on attention-deficit/hyperactivity disorder common psychiatric comorbidities: mechanistic insights on binge eating disorder and depression

**DOI:** 10.3389/fnins.2023.1118253

**Published:** 2023-06-30

**Authors:** José Ramón Gutiérrez-Casares, Cristina Segú-Vergés, Juncal Sabate Chueca, Tamara Pozo-Rubio, Mireia Coma, Carmen Montoto, Javier Quintero

**Affiliations:** ^1^Unidad Ambulatoria de Psiquiatría y Salud Mental de la Infancia, Niñez y Adolescencia, Hospital Perpetuo Socorro, Badajoz, Spain; ^2^Anaxomics Biotech, Barcelona, Spain; ^3^Research Programme on Biomedical Informatics (GRIB), Departament de Ciències Experimentals i de la Salut, Universitat Pompeu Fabra, Barcelona, Spain; ^4^Department of Medical, Takeda Farmacéutica España, Madrid, Spain; ^5^Servicio de Psiquiatría, Hospital Universitario Infanta Leonor, Departamento de Medicina Legal, Patología y Psiquiatría, Facultad de Medicina, Universidad Complutense de Madrid, Madrid, Spain

**Keywords:** attention-deficit/hyperactivity disorder, lisdexamfetamine, mathematical modeling, binge eating disorder, depression

## Abstract

Attention-deficit/hyperactivity disorder (ADHD) is a psychiatric condition well recognized in the pediatric population that can persist into adulthood. The vast majority of patients with ADHD present psychiatric comorbidities that have been suggested to share, to some extent, the pathophysiological mechanism of ADHD. Lisdexamfetamine (LDX) is a stimulant prodrug approved for treating ADHD and, in the US, also for binge eating disorder (BED). Herein, we evaluated, through a systems biology-based *in silico* method, the efficacy of a virtual model of LDX (vLDX) as ADHD treatment to improve five common ADHD psychiatric comorbidities in adults and children, and we explored the molecular mechanisms behind LDX’s predicted efficacy. After the molecular characterization of vLDX and the comorbidities (anxiety, BED, bipolar disorder, depression, and tics disorder), we created a protein-protein interaction human network to which we applied artificial neural networks (ANN) algorithms. We also generated virtual populations of adults and children-adolescents totaling 2,600 individuals and obtained the predicted protein activity from Therapeutic Performance Mapping System models. The latter showed that ADHD molecular description shared 53% of its protein effectors with at least one studied psychiatric comorbidity. According to the ANN analysis, proteins targeted by vLDX are predicted to have a high probability of being related to BED and depression. In BED, vLDX was modeled to act upon neurotransmission and neuroplasticity regulators, and, in depression, vLDX regulated the hypothalamic-pituitary-adrenal axis, neuroinflammation, oxidative stress, and glutamatergic excitotoxicity. In conclusion, our modeling results, despite their limitations and although requiring *in vitro* or *in vivo* validation, could supplement the design of preclinical and potentially clinical studies that investigate treatment for patients with ADHD with psychiatric comorbidities, especially from a molecular point of view.

## 1. Introduction

Attention-deficit/hyperactivity disorder (ADHD) is an impairing psychiatric condition affecting children and adults and is characterized by symptoms of inattention, hyperactivity, and impulsivity (Fayyad et al., 2007; Thomas et al., 2015; Caye et al., 2019). Noteworthy, 80% of adult (Mannuzza et al., 1998; Biederman, 2004; McGough and Barkley, 2004; Wilens et al., 2004; Katzman et al., 2017; Ohnishi et al., 2019; Howard et al., 2020; Kittel-Schneider and Reif, 2020) and 67% of pediatric-adolescent (Larson et al., 2011) patients with ADHD presents psychiatric comorbidities, including depression, anxiety, substance abuse, learning and coordination disorders, and conduct disorders (Mannuzza et al., 1998; Wilens et al., 2004). In ADHD adult patients, the most common psychiatric comorbidities are depression, substance-related disorders, anxiety, and eating disorders (Wilens et al., 2004; Sobanski, 2006; Sobanski et al., 2007; Ohnishi et al., 2019). On the other hand, children and adolescents with ADHD present more often learning and coordination disorders and conduct disorders (including oppositional defiant disorder) but also anxiety and depression (Gillberg et al., 2004; Joelsson et al., 2016; Cuffe et al., 2020). Since ADHD symptoms and its psychiatric comorbidities share similarities, a partial overlap of their pathophysiological mechanisms has been suggested (Franke et al., 2012; Lee et al., 2013; Katzman et al., 2017; Laas et al., 2017).

Currently, a combination of both pharmacological and psychotherapeutic treatments such as cognitive-behavioral therapy (CBT) are treatment options in the clinic for children, adolescents and adults patients with ADHD (Coelho et al., 2017; Pan et al., 2019). CBT has been regarded as one of the most effective psychological treatments for ADHD and, when combined with medication, is associated with greater improvements in adherence to treatment and in core ADHD symptoms, social functions and comorbid symptoms (Coelho et al., 2017; Pan et al., 2019). On the other hand, ADHD medication includes stimulants, among which lisdexamfetamine (LDX), a prodrug with proven efficacy for treating binge eating disorder (BED) and approved for this indication in the adult population of the US (U. S. Food and Drug Administration, 2017). However, the efficacy of LDX on patients with ADHD and psychiatric comorbidities has been poorly studied (Kollins et al., 2011; Roncero and Álvarez, 2014). Also, mechanistic studies on LDX are scarce and could inform clinicians and improve clinical trial designs (Hutson et al., 2014). In this sense, systems biology methods have aided in untangling the molecular effects of drugs in complex clinical settings, such as treatment-resistant pathologies (Akil et al., 2018), data analysis for personalized medicine (Davis et al., 2019), mechanistic studies in drug-refractory patients (Lorén et al., 2019), and *in silico* head-to-head trials (Caye et al., 2019). We recently performed *in silico* studies between virtual models of LDX (vLDX) and methylphenidate to treat ADHD in adults and children (Gutiérrez-Casares et al., 2021; Gutierrez-Casares et al., 2023). As an extension of that study, we considered essential to investigate the effects of vLDX over ADHD common psychiatric comorbidities in patients with ADHD.

The main objective of our study was to evaluate, through a systems biology-based *in silico* method, the efficacy of vLDX as ADHD treatment to improve some common ADHD psychiatric comorbidities. In addition, we explored the molecular mechanisms behind vLDX’s predicted efficacy in virtual adult and pediatric-adolescent comorbid patients with ADHD.

## 2. Materials and methods

### 2.1. Study design

In this study, we applied systems biology-based *in silico* approaches to evaluate the potential relationship of LDX with psychiatric comorbidities by applying Therapeutic Performance Mapping System (TPMS) technology (Jorba et al., 2020). TPMS is a computational tool that models the protein pathways of a drug or pathology explaining a clinical outcome or phenotype. We applied this technology using two complementary approaches. First, we used artificial neural network (ANN) (Artigas et al., 2020) algorithms, which provide predictive information. Then, we used sampling-based methods combined with other modeling approaches to obtain quantitative systems pharmacology (QSP)-based virtual patient models to explore the mechanisms behind the predicted relationships, as extensively described previously (Gutiérrez-Casares et al., 2021). Briefly, our QSP approach included: the generation of a virtual randomized population following randomized clinical trials’ (Ginsberg et al., 2011; Retz et al., 2012; Coghill et al., 2013) characteristics and European reference population distribution (de Onis et al., 2007; The European Social Survey, 2018), a physiologically based pharmacokinetic (PBPK) modeling approach based on a 14-compartment model (Peters, 2008; Rostami-Hodjegan, 2012; Ciffroy et al., 2016; Brochot and Quindroit, 2018), and TPMS systems biology-based modeling (Jorba et al., 2020), which mimics the human pathophysiology at a protein-network level using machine learning. This method provided virtual patient-specific QSP models of virtual drugs (i.e., defined by the model from available literature) that complied with the accuracy and quality measurements set for each step (Gutiérrez-Casares et al., 2021).

### 2.2. Characterization of ADHD, comorbidities, and vLDX

We undertook a bibliographically based structured search to obtain a protein-based molecular characterization of ADHD and of LDX to be used as input for TPMS models (Jorba et al., 2020). Aside from a review of official regulatory documentation and drug-target dedicated databases, a review of the currently available bibliography regarding known targets of the drugs was performed in PubMed on April 27, 2020. The specific searches performed can be found in Gutiérrez-Casares et al. (2021). The list of publications identified in the specific searches was retrieved and assessed at the title and abstract level. If molecular information describing pathophysiological conditions was found, the full texts were thoroughly reviewed to identify the main pathophysiological processes known to be involved in the diseases. We defined ADHD by four motives (or biological processes) that describe the pathophysiology of the disease: neurotransmitter imbalance, neuroinflammation, circadian system imbalance, and altered neural viability. We only included proteins for which a functional role on the disease was reported (Supplementary Table A). Then, we applied the same procedure to characterize all five studied comorbidities, i.e., anxiety, BED, bipolar disorder, depression, and tics disorder (Supplementary Table A).

For the definition and modeling of the virtual drug vLDX, we considered its prodrug nature, and compiled information and modeled the behavior of its active metabolite, d-amphetamine, as previously described (Gutiérrez-Casares et al., 2021). Briefly, we defined vLDX by the protein targets affected by LDX active form, according to a structured search bibliographical analysis, and considered as drug targets those proteins for which the drug had activity either *in vitro* or *in vivo* (Table 1). For the QSP modeling, we used d-amphetamine concentration curve associated to LDX treatment (Gutiérrez-Casares et al., 2021), obtained from adjusting a physiologically based pharmacokinetic model (Peters, 2008; Rostami-Hodjegan, 2012; Ciffroy et al., 2016; Brochot and Quindroit, 2018) to reported pharmacokinetic parameters (oral administration; kidney as main clearance organ; bioavailability: 96.4%) and real plasma concentration data (Krishnan and Zhang, 2008; Boellner et al., 2010; Maldonado, 2013; Bundesinstitut für Arzeimittel und Medizinprodukte, 2020). This approach allowed us to obtain a drug concentration curve per virtual patient considering their individual characteristics (weight, height, age, and sex).

**TABLE 1:** Identified protein targets for lisdexamfetamine (Gutiérrez-Casares et al., 2021).

Gene name	Protein name	Effect*	Reference of LDX target
TAAR1	Trace amine-associated receptor 1	1	Ekstrand et al., 2019
SLC18A2	Synaptic vesicular amine transporter (VMAT2)	−1	Hutson et al., 2014; Ekstrand et al., 2019
SLC6A3	Sodium-dependent dopamine transporter (DAT)	−1	Cheney et al., 2014; Hutson et al., 2014; Strajhar et al., 2019
SLC6A2	Sodium-dependent noradrenaline transporter (NET)	−1	Cheney et al., 2014; Hutson et al., 2014
SLC6A4	Sodium-dependent serotonin transporter (SERT)	−1	Hutson et al., 2014
MAOA	Amine oxidase (flavin-containing) A	−1	Dew and Kollins, 2010; Cheney et al., 2014
MAOB	Amine oxidase (flavin-containing) B	−1	Dew and Kollins, 2010; Cheney et al., 2014

*Effect refers to the drug’s action on the protein, 1 denotes activation of protein function, −1 inhibition of protein function. LDX, lisdexamfetamine.

### 2.3. Therapeutic performance mapping system

Therapeutic performance mapping system is based on the human protein interaction network and pharmacology and pathophysiology information, which constitutes the training set (Supplementary Table B). To create TPMS models, we exploited an in-house database drawn from public sources—KEGG (Kanehisa et al., 2017), REACTOME (Jassal et al., 2020), INTACT (Orchard et al., 2014), BIOGRID (Oughtred et al., 2019), HPRD (Keshava Prasad et al., 2009), and TRRUST (Han et al., 2018)—as a basis to create the protein-protein interaction human network. We used Cytoscape v. 3.6.0 (Shannon et al., 2003) to visualize this network. We applied ANN (Artigas et al., 2020) algorithms that identify the probability of a specific relationship between two or more protein sets. The ANN algorithm provides a score (ranging from 0 to 100%) associated with a probability (*p*-value) that the drug (group of protein targets) and the pathology (defined by the molecular description of pathological processes detected in the characterization) being evaluated are functionally connected. Therefore, scores greater than 92% indicate a very strong relationship with a *p*-value below 0.01; scores between 77 and 92% imply a strong relationship with a *p*-value between 0.01 and 0.05; scores between 37 and 77% have a medium relationship and a *p*-value in the range 0.05–0.25; and scores lower than 37% indicate a poor relationship with *p*-values above 0.25. Finally, we modeled the mechanisms of vLDX for the most relevant comorbidities by using sampling methods after QSP modeling. These models provide mechanisms of action that explain how a stimulus (i.e., proteins activated or inhibited by a drug) produces a response (i.e., proteins active or inhibited in a phenotype) (Jorba et al., 2020). The quality of these models is measured by its accuracy, which is calculated as the percentage of compliance of all drug-pathophysiology relationships included in the training set (Jorba et al., 2020; Gutiérrez-Casares et al., 2021).

### 2.4. Participants: QSP modeled virtual patients

The methodology used for developing the *in silico* clinical trial from which this detailed mechanistic study derives is described in detail elsewhere (Gutiérrez-Casares et al., 2021). Briefly in that study, we used expression data to explore molecular variability and estimate a minimum population size to ensure statistical power; 71 was set as the minimum population size per cohort. Therefore, we generated two virtual populations (adults–18 years old and older–and children-adolescents–from 6 to 18 years old) with ADHD comorbidities, with 100 virtual patients per each age category and comorbidity.

We created a randomized population demographic characteristics using ADHD trials as a reference for mean demographic values (Ginsberg et al., 2011; Retz et al., 2012; Coghill et al., 2013) and standard population information (de Onis et al., 2007; The European Social Survey, 2018) to obtain the randomized patient distribution fitting the desired population values.

Quantitative systems pharmacology models are generated following the TPMS methodology but incorporating drug concentration data at different timepoints in addition to molecular inputs, which add patient-specific quantitative data. To this end, a set of drug concentration timepoints in the target tissue—brain in this study—can be associated with the modulation of the drug’s target proteins. Accordingly, the resulting LDX drug’s target modulation-efficacy relationships were used as extra parameters in the TPMS training set, resulting in the final QSP models. Through the use of clinical efficacy values for various drugs tested in ADHD clinical trials, modeled drug concentration curves were plotted and used to obtain restrictions on target inhibition with ADHD modulation. These restrictions were compatible with systems biology-based TPMS models (Jorba et al., 2020) and conferred them a quantitative dimension. Thus, we obtained a TPMS-derived QSP model per each virtual patient and each virtual drug. We used comorbidity-specific virtual patients for sampling methods-based mechanistic evaluation in each case.

### 2.5. Outcomes and measures of the QSP models

Due to the systems-biology–based nature of the virtual patients’ resulting models, all measures were centered on protein activity. We obtained the predicted protein activity (ranging from −1 to 1) from each QSP model (Jorba et al., 2020; Gutiérrez-Casares et al., 2021). We analyzed these data for each protein individually, calculated the tSignal (Jorba et al., 2020; Gutiérrez-Casares et al., 2021), and defined a reverted protein as one whose activation sign in the comorbidity under study was reverted due to the effect of vLDX. In the current study, we considered reverted proteins as those for which (A) the direction of their activation was opposite to the one to induce the disease and (B) the absolute value of its predicted protein activity was at least half of the maximum value achievable (i.e., higher than 0.5). For mechanisms of action, exploration, we focused on the paths (three-step protein interaction between the stimulus and the response) obtained from TPMS models, as previously described (Jorba et al., 2020; Segú-Vergés et al., 2021), that (A) justified at least a protein effector of the studied comorbidity reverted by vLDX with at least 80% of the maximum value achievable (i.e., | predicted activation| > 0.8) and (B) with a frequency = 100% within the population of comorbidity-specific virtual patients.

## 3. Results

### 3.1. ADHD—Psychiatric comorbidities protein-protein interactome

**FIGURE 1:**
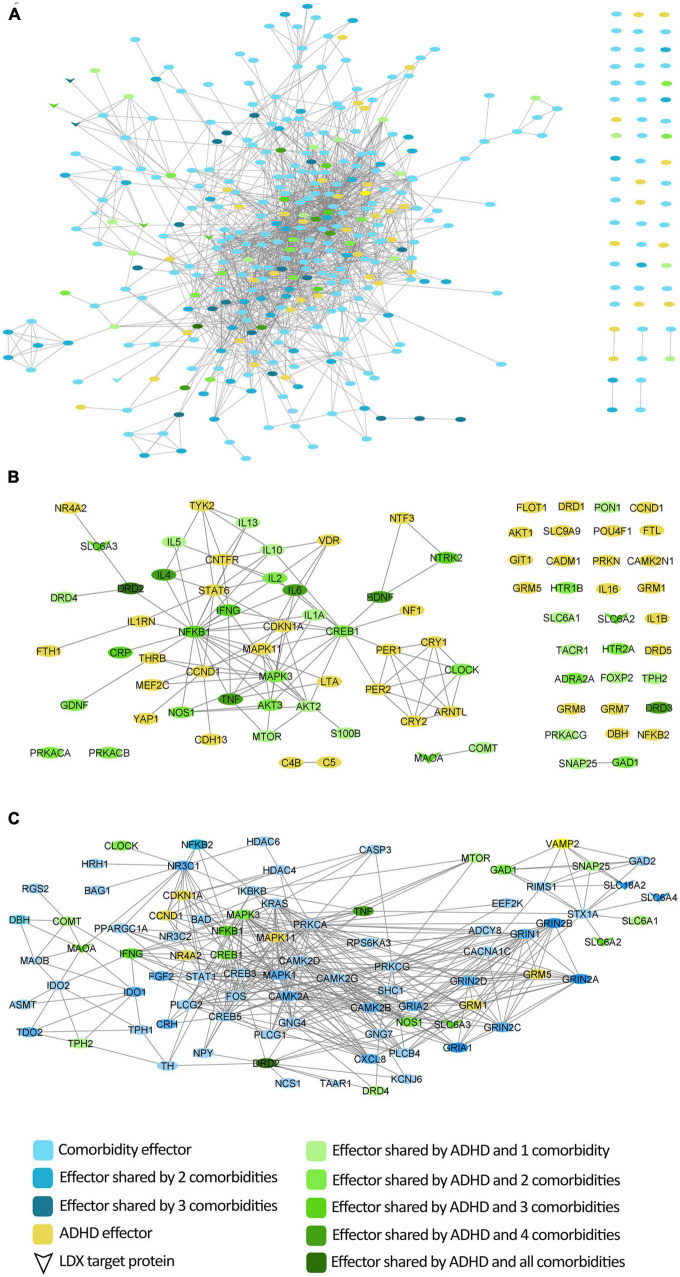
Protein interactome around ADHD molecular characterization considering the protein effectors of its most common comorbidities. **(A)** Network of direct interactions between the protein effectors of ADHD and those of its comorbidities; **(B)** Network of ADHD protein effectors representing the overlap with comorbidities; **(C)** Network of vLDX targets and their interactions with the protein effectors of ADHD and its comorbidities. ADHD, attention-deficit/hyperactivity disorder; vLDX, virtual lisdexamfetamine.

According to the bibliography-based molecular characterization of the pathologies under study (Supplementary Table A), the ADHD molecular description shared 53% of its protein effectors with at least one studied psychiatric comorbidity (Figures 1A, B). ADHD shared between 8 and 34% of its effectors with the molecular definition of each individual comorbidity, being anxiety the one with the largest overlap. According to the protein-protein network, 84% of ADHD molecular effectors were shared or directly connected to effectors of at least one comorbidity. The comorbidity with the largest connection was depression, for which 52% of ADHD effectors were shared or directly connected to 54% of depression effectors. All vLDX targeted proteins were found to be effectors of ADHD or at least one of its psychiatric comorbidities (Figure 1C). Interestingly, the LDX target TAAR1, classified as a BED effector, is directly related to DRD2, a protein identified as an effector in ADHD and all five characterized psychiatric comorbidities.

### 3.2. ANN-based efficacy evaluation of vLDX over ADHD comorbidities

Artificial neural networks showed that the proteins targeted by vLDX had a high probability (*p* < 0.01) of being related to BED and depression (Table 2). However, this probability was moderate (*p* < 0.25) for anxiety and tic disorders and very low (*p* ≥ 0.25) for bipolar disorder. Although the ANN values for anxiety (76%) and tic disorders (71%) were very close to the *p*-value < 0.05 threshold (77%), only the results for BED and depression were considered statistically significant and subjected to detailed mechanistic evaluation.

**TABLE 2:** Artificial neural network evaluation of the impact of virtual lisdexamfetamine over comorbidities.

	Depression	Anxiety	Bipolar disorder	Binge eating disorder	Tic disorder
vLDX	Very high (94%)	Medium (76%)	Low (17%)	Very high (94%)	Medium (71%)

Categories: very high (score [100–92], *p*-value < 0.01); high (score [92–77], *p*-value < 0.05); medium (score [77–37], *p*-value < 0.25); low (score < 37, *p*-value ≥ 0.25). vLDX, virtual lisdexamfetamine.

### 3.3. Mechanism of action of vLDX in BED as ADHD comorbidity

**FIGURE 2:**
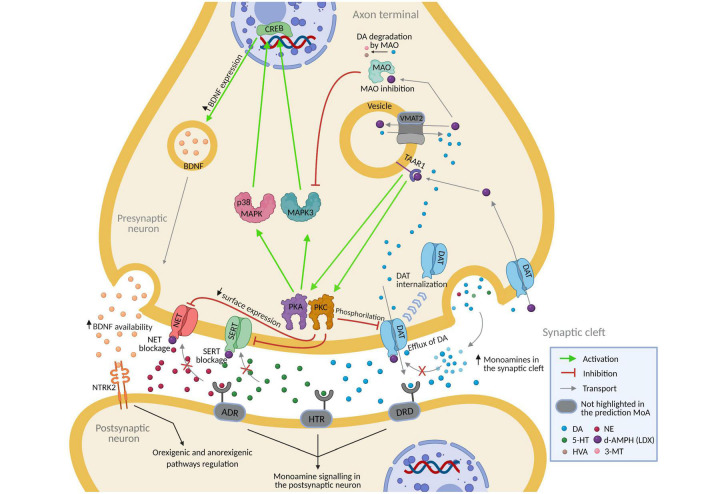
Predicted mechanism of action of lisdexamfetamine (according to vLDX QSP models) over binge eating disorder in patients with ADHD presenting this comorbidity. Supplementary Figure A and Supplementary Table C contain the sources of information found in the scientific literature supporting the predicted mechanism. Created with BioRender.com. 3-MT, 3-Methoxytyramine; 5-HT, Serotonin; ADHD, attention-deficit/hyperactivity disorder; ADR, adrenoreceptors; DA, dopamine; d-AMPH, d-Amphetamine; DAT, sodium-dependent dopamine transporter; DRD, dopamine receptor; HTR, serotonin receptor; HVA, homovanillic acid; NE, norepinephrine; NET, sodium-dependent noradrenaline transporter; NTRK2, neurotrophic receptor tyrosine kinase 2; QSP, quantitative systems pharmacology; SERT, sodium-dependent serotonin transporter; vLDX, virtual lisdexamfetamine.

Virtual model of Lisdexamfetamine modulated BED by regulating neurotransmission (DAT, MAO, NET1) and neuronal survival and plasticity (BDNF-NTRK2) (Figure 2). In both adult and children-adolescent models, vLDX was able to revert eight BED-related protein effectors with | predicted protein activity| > 0.5, four of which (NET1, DAT1, BDNF, and NTRK2) are also involved in ADHD pathophysiological mechanisms (Table 3). In patients with ADHD presenting BED as a comorbidity, the protein targets DAT1, NET1, SERT, and TAAR1 were highly reverted by vLDX, as well as the neurotrophic factor BDNF (affecting the BDNF-NTRK2 pathway) (Figure 2, Supplementary Figure A and Supplementary Table C). Interestingly, NET1 was predicted to be inhibited by vLDX not only directly but also indirectly through TAAR1 activation, inducing the PKC signaling pathway. In addition, BDNF and its receptor NTRK2 were activated via TAAR1-initiated PKA signaling. vLDX was also found to modulate CRHR1 (Table 3).

**TABLE 3:** Proteins related to binge eating disorder and reverted by virtual lisdexamfetamine with | predicted protein activity| > 0.5 in both adult and pediatric-adolescent populations.

Gene name	Motives in BED	ADHD effector
BDNF	Neurotransmission alteration in impulse control circuitry	Yes
CRHR1	Deregulation of appetite mechanisms	No
NTRK2	Neurotransmission alteration in impulse control circuitry	Yes
SLC18A2/VMAT2	Neurotransmission alteration in impulse control circuitry	No
SLC6A2/NET1	Neurotransmission alteration in impulse control circuitry	Yes
SLC6A3/DAT1	Neurotransmission alteration in impulse control circuitry	Yes
SLC6A4/SERT	Neurotransmission alteration in impulse control circuitry	No
TAAR1	Neurotransmission alteration in impulse control circuitry	No

ADHD, attention-deficit/hyperactivity disorder; BED, binge eating disorder.

### 3.4. Mechanism of action of vLDX in depression as ADHD comorbidity

**FIGURE 3:**
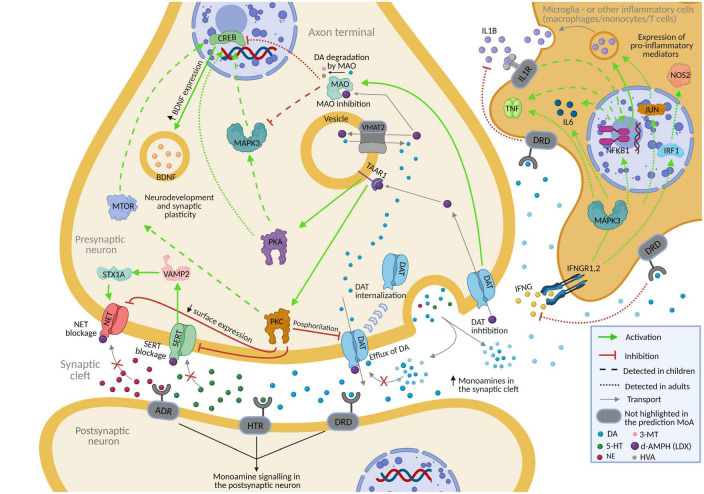
Predicted mechanism of action of lisdexamfetamine (according to vLDX QSP models) over depression in patients with ADHD presenting this comorbidity. Supplementary Figure B and Supplementary Table D contain the sources of information found in the scientific literature supporting the predicted mechanism. Created with BioRender.com. 3-MT, 3-Methoxytyramine; 5-HT, serotonin; ADHD, attention-deficit/hyperactivity disorder; ADR, adrenoreceptor; DA, dopamine; d-AMPH, d-Amphetamine; DAT, sodium-dependent dopamine transporter; DRD, dopamine receptor; HTR, serotonin receptor; HVA, homovanillic acid; IL1R, interleukin-1 receptor; IFNG, interferon-gamma; IFNGR1.2, interferon-gamma receptor; NE, norepinephrine; NET, sodium-dependent noradrenaline transporter; QSP, quantitative systems pharmacology; SERT, sodium-dependent serotonin transporter; vLDX, virtual lisdexamfetamine.

Virtual model of Lisdexamfetamine modulated depression by regulating glutamatergic excitotoxicity, neurotransmission, neuroplasticity, the hypothalamic-pituitary-adrenal (HPA) axis, neuroinflammation, and oxidative stress (Table 4). According to our models, vLDX was able to revert 49 depression-related effectors in adults and 44 in children with | predicted protein activity| > 0.5, 17 of which (BDNF, CREB1, CRP, IL-1A, IL-1B, IL-6, MAO, MTOR, NFKB1, NTRK2, PRKACA, PRKACB, NET1, DAT1, and TNF in both populations and IFNG and IL-2 only in adults) were also involved in ADHD pathophysiological mechanisms. In addition, in both adult and pediatric-adolescent ADHD patient populations, vLDX was found to modulate depression-related processes through glutamatergic excitotoxicity (VAMP2), neurotransmission alteration (SERT, DAT1, MAO, NET1, NOS2, TAAR1, IFN, IFNGR1, IFNGR2), HPA axis hyperactivation (BDNF), neuroinflammation (TNF, IL-1, IL-6, IFNG, NF-κB), and loss of neural plasticity (CREB1, BDNF, PKA) (Figure 3, Supplementary Figure B and Supplementary Table D).

**TABLE 4:** Depression-related proteins reverted by virtual lisdexamfetamine with | predicted protein activity| > 0.5 in adult, pediatric-adolescent or both virtual populations.

Gene name	Motives in depression	ADHD effector	Population
BDNF	HPA axis hyperactivation/Loss of neural plasticity and Neurogenesis	Yes	Both
CCL2	Neuroinflammation	No	Both
CREB1	Loss of neural plasticity and neurogenesis	Yes	Both
CRH	HPA axis hyperactivation/Neuroinflammation	No	Both
CRHR1	HPA axis hyperactivation	No	Both
CRP	Neuroinflammation	Yes	Both
CXCL8	Neuroinflammation	No	Both
DRD2	Neurotransmission alteration	Yes	Both
EEF2K	Loss of neural plasticity and neurogenesis	No	Both
FGF1	Loss of neural plasticity and Neurogenesis/Neuroinflammation	No	Both
GSK3A	Loss of neural plasticity and neurogenesis	No	Both
GSK3B	Loss of neural plasticity and neurogenesis	No	Adult
HTR1A	Neurotransmission alteration	No	Both
IFNG	Neuroinflammation	Yes	Adult
IFNGR1	Neurotransmission alteration	No	Both
IFNGR2	Neurotransmission alteration	No	Both
IKBKB	Loss of neural plasticity and neurogenesis	No	Both
IL18	Neuroinflammation	No	Both
IL1A	Neuroinflammation	Yes	Both
IL1B	Neuroinflammation	Yes	Both
IL2	Neuroinflammation	Yes	Adult
IL6	Neuroinflammation	Yes	Both
MAO	Neurotransmission alteration	Yes	Both
MMP2	Neuroinflammation	No	Both
MTOR	Loss of neural plasticity and neurogenesis	Yes	Both
NFKB1	Loss of neural plasticity and Neurogenesis/Neuroinflammation	Yes	Both
NLRP3	Neuroinflammation	No	Both
NOS2	Neurotransmission alteration	No	Both
NR3C1	HPA axis hyperactivation	No	Both
NTRK2	HPA axis hyperactivation	Yes	Both
POMC	HPA axis hyperactivation	No	Both
PPARGC1A	Glutamatergic excitotoxicity/Neuroinflammation	No	Both
PRKACA	Loss of neural plasticity and neurogenesis	Yes	Both
PRKACB	Loss of neural plasticity and neurogenesis	Yes	Both
RAC1	Loss of neural plasticity and neurogenesis	No	Both
RPS6KB1	Loss of neural plasticity and neurogenesis	No	Both
RPS6KB2	Loss of neural plasticity and neurogenesis	No	Both
SERPINE1	Loss of neural plasticity and neurogenesis	No	Both
SLC6A2/NET1	Neurotransmission alteration	Yes	Both
SLC6A3/DAT1	Neurotransmission alteration	Yes	Both
SLC6A4/SERT	Neurotransmission alteration	No	Both
SMAD2	Neuroinflammation	No	Both
SMAD4	Neuroinflammation	No	Adult
SOD1	Oxidative stress	No	Adult
SOD2	Oxidative stress	No	Both
STAT1	Neuroinflammation	No	Both
TIMP2	Neuroinflammation	No	Both
TNF	HPA axis hyperactivation/Neuroinflammation	Yes	Both
VAMP2	Glutamatergic excitotoxicity	No	Both

ADHD, attention-deficit/hyperactivity disorder; HPA, hypothalamic-pituitary-adrenal.

## 4. Discussion

Through a systems biology-based *in silico* method, we found that vLDX’s target proteins were related to BED and depression. Considering LDX use as treatment in patients with ADHD and taking a previously described virtual population of patients with ADHD and psychiatric comorbidities (Gutiérrez-Casares et al., 2021), we explored the detailed mechanisms behind vLDX activity on BED and depression as comorbidities in patients with ADHD. According to our modeling results, in BED, vLDX was shown to act upon neurotransmission and neuroplasticity regulators, and, in depression, vLDX regulated the HPA axis, neuroinflammation, oxidative stress, and glutamatergic excitotoxicity.

Based on the available literature, the diseases’ molecular characterization showed that more than half of the proteins affected by ADHD were also deregulated in at least one of the evaluated psychiatric comorbidities (i.e., depression, anxiety, BED, bipolar disorder, and tic disorders). In adults, ADHD is highly comorbid with other psychiatric disorders; 65–89% of patients with ADHD present at least one psychiatric comorbidity, mainly anxiety and mood and substance use disorders (Biederman, 2004; Kessler et al., 2006; Sobanski, 2006; Sobanski et al., 2007; Piñeiro-Dieguez et al., 2016). In children and adolescents with ADHD, psychiatric comorbidities are even more prevalent and 60–100% present at least one psychiatric comorbidity, mostly conduct and anxiety disorders and depression (Biederman, 2004; Sobanski, 2006; Sobanski et al., 2007; Joelsson et al., 2016; Cuffe et al., 2020).

The association between ADHD and BED has been established by several clinical studies in adults and children (Cortese et al., 2007; Bleck et al., 2015; Rankin et al., 2016). In our ANN analysis, vLDX pharmacological protein targets were most likely related to BED development, thus supporting LDX’s ability to manage BED. These results are supported by several randomized, double-blind, placebo-controlled multicenter studies that have demonstrated the efficacy of LDX in treating BED (McElroy et al., 2016; Hudson et al., 2017). Of note, in the US, LDX is indicated for BED treatment in adults (U. S. Food and Drug Administration, 2017). Although not approved for use in children and adolescents with this indication, LDX has proven to be effective in adolescents >12 years old (Guerdjikova et al., 2019; Srivastava et al., 2019).

Monoamine transporters NET1, DAT1, and SERT, which are reported LDX targets (Popovic et al., 2009; Guerdjikova et al., 2016), were predicted to be related to vLDX mechanisms in BED and depression improvement in ADHD virtual patients. In our sampling methods-based BED model, NET1 was also inhibited indirectly via the PKC signaling pathway initiated by vLDX-mediated TAAR1 stimulation, which has been reported to improve neurotransmission alteration (Xie and Miller, 2007; Bermingham and Blakely, 2016; Appolinario et al., 2019). Similarly, the BDNF-NTRK2 pathway was activated through TAAR1-initiated PKA signaling, which has been reported to enhance neurotransmission (Xue et al., 2016; Ceccarini et al., 2020). Notably, BDNF synthesis and secretion has been located in presynaptic neuronal sites (Song et al., 2017). vLDX was also found to modulate CRHR1, a protein that has been reported to be involved in appetite regulation (Ghitza et al., 2006; Iemolo et al., 2013; Micioni Di Bonaventura et al., 2017, 2020, 2021). A recent review (Schneider et al., 2021) concluded that LDX’s mechanisms to treat BED encompass a complex combination of processes, including effects on appetite/satiety, reward, and cognitive processes (such as attention and impulsivity/inhibition), mainly through catecholamine and serotonin neuronal pathways in the brain, which are in line with our results. This study also evidenced a lack of specific information on mechanisms that could be differential between both populations. According to our results, no differences in the mechanisms reducing BED were found between our two virtual populations. However, as further discussed in detail later in this section, our models only considered age differences derived from PBPK modeling and brain drug concentration curves, which rendered our models incomplete in this sense.

Our ANN analysis also showed a highly probable relation between proteins targeted by vLDX and depression. In this sense, current available evidence in the literature points toward a slight improvement in depressive symptoms when using LDX as antidepressant augmentation therapy (Corp et al., 2014; Madhoo et al., 2014; Giacobbe et al., 2018). In our study, vLDX was found to act upon DAT1, MAO (Popovic et al., 2009), TAAR1 (Moore et al., 2018), NET1, SERT (Guerdjikova et al., 2016), and NOS2 (Inserra et al., 2019), all of which have been linked with the characteristic impairment in neurotransmission observed in depressive patients (Sung et al., 2003; Sotnikova et al., 2009; Gaweska and Fitzpatrick, 2011; Vaughan and Foster, 2013; Moore et al., 2018; Inserra et al., 2019; Kegg Pathway Database, 2020). Interestingly, DAT1 inhibition has been shown to prevent dopamine reuptake into the cytosol (Vaughan and Foster, 2013) and its degradation by MAO (Gaweska and Fitzpatrick, 2011; Kegg Pathway Database, 2020), hence, improving the impaired neurotransmission (Miller and Raison, 2016). Also, TAAR1 may directly interact with DAT, thereby affecting dopamine neurotransmission via modulation of DAT function (Sotnikova et al., 2009). The inhibition of NET1 by vLDX via TAAR1 stimulation could ameliorate the neurotransmission alteration found in depressive patients (Peterson et al., 2015; Bermingham and Blakely, 2016; Moore et al., 2018). Moreover, this effect on NET1 may be enhanced by the blockade of STX1A by vLDX through SERT since it supports surface trafficking of NET1 and regulates the transporter catalytic function (Sung et al., 2003). On the other hand, the reduction in NOS2 levels exerted by LDX could permit the production of norepinephrine, usually blocked by this enzyme in depression (Inserra et al., 2019). According to our results, vLDX affected processes directly related to the HPA axis, whose hyperactivity has been implicated in the pathophysiology of depression (Pariante and Lightman, 2008; Keller et al., 2017; Menke, 2019; Iob et al., 2020; Pitsillou et al., 2020). Besides, vLDX modulated proteins involved in neuroinflammation and oxidative stress in depressive states (Inserra et al., 2019; Pitsillou et al., 2020), processes that have been repeatedly suggested to play a role in these patients (Yanik et al., 2004; Bakunina et al., 2015; Black et al., 2015; Liu et al., 2015; Lindqvist et al., 2017). According to our model, and as previously described in experimental models (Vidal and Pacheco, 2020), dopamine signaling modulated neuroinflammation by regulating different immune cells, including microglia, cells from the monocytic lineage, and T cells. High dopamine levels have been reported to reduce nitric oxide production in rodent microglial cells (Katrin et al., 2005). In relation to neuroinflammation, by regulating dopamine synaptic availability, vLDX could modulate a wide spectrum of cytokines and other inflammatory factors induced by microglia (or other immune cells) such as IL-6 (Garner et al., 2018; Bobbo et al., 2019), IL-1B (Liu and Quan, 2018), TNF (Welser-Alves and Milner, 2013), and IFNG receptors (Tsuda et al., 2009; Ta et al., 2019), all shown to be involved in the inflammatory process of depression (Inserra et al., 2019; Pitsillou et al., 2020). Particularly, the current corpus of evidence points toward a role for high dopamine levels in attenuating the inflammatory activation of microglia through the stimulation of low-affinity dopamine receptors (including DRD1, DRD2, and DRD4) (Vidal and Pacheco, 2020). Our model also showed that vLDX activated BDNF, an essential factor for neurogenesis and neural plasticity and downregulated in depression (Juhasz et al., 2011; Schmid et al., 2015; Cui et al., 2019; Underhill et al., 2021). BDNF activation by CREB1 (Juhasz et al., 2011) might be possible because of the inhibition of DAT1 and MAO by vLDX or the stimulation of PRKACB by TAAR1 (Schmid et al., 2015; Underhill et al., 2021). In addition, we found that vLDX dampened glutamatergic excitotoxicity through VAMP2 modulation, a phenomenon that has been observed in patients with depression (Mitchell and Baker, 2010; Dantzer and Walker, 2014; Miller and Raison, 2016; Cui et al., 2019; Pitsillou et al., 2020). In summary, our models propose several mechanisms involved in the modulation of depression-related processes by vLDX, including, the regulation of the HPA axis, oxidative stress and neuroinflammation, neural plasticity and glutamatergic neurotoxicity, mainly through the joint effect over neurotransmitter transporters (SERT, DAT1 and NET) but also regulation of intracellular PKA and PKC signaling through TAAR1 agonism. Finally, some minor differences were observed between adult and children depression models, which rise from the differences in drug pharmacokinetics in both populations according to physiologically based pharmacokinetic models (Ciffroy et al., 2016; Brochot and Quindroit, 2018; Gutiérrez-Casares et al., 2021). Further studies considering other factors (such as neurodevelopment with age) might provide more specific insights into the different mechanisms of LDX in adults regarding pediatric patients.

Notwithstanding the abovementioned, our study presented some limitations that should also be considered. Firstly, the main constraint was the available information, at the moment of the study, on the drug and diseases under study upon which our models were built. In the current study, this factor might be aggravated by the difficulty of studying psychiatric diseases molecularly and by the high genetic and signaling overlap between ADHD and its psychiatric comorbidities (Faraone and Mick, 2010; Faraone and Larsson, 2019), which may act as confounding factors at the clinical and molecular levels. Thus, our models could have been susceptible to information availability bias, leading to some aspects being overlooked. This information could certainly be more extensive in the future (e.g., unknown drug targets or yet undescribed pathophysiological processes), circumscribing our analysis to the present time. Additionally, our modeling methodology has the inherent restraints of mathematical modeling, which cannot fit 100% of the training data information (TPMS viable solutions have always at least 85% accuracy, easily achieving around 95% mean accuracy). Secondly, our approach considered only the impact of demographic characteristics, including age, on the PBPK modeling. However, psychiatric conditions can involve different neuronal mechanisms depending on the patient’s neurodevelopmental stage (Sonuga-Barke and Halperin, 2010; Dreyfuss et al., 2017), which our models did not contemplate. This limitation could prevent the detection of relevant results regarding differences in the mechanisms mediating the effects of LDX in adults and pediatric and/or adolescent populations. These and other limitations of the QSP modeling and their impact on the *in silico* clinical trial approach are described in detail elsewhere (Gutiérrez-Casares et al., 2021). Third, our approach is a simplification of the complex real-world clinical and pharmacological context of ADHD patients and we did not consider the outcomes in patients taking other substances. Individuals with ADHD are known to self-medicate with substances such as nicotine and cannabinoids because they could alleviate ADHD symptoms (and its comorbidities) (Lee et al., 2011). Although patients with ADHD have regularly stated that cannabis has helped to improve their ADHD symptoms, it is still controversial how and whether cannabis impacts on ADHD symptoms or on pharmacological therapies (Francisco et al., 2023). Regarding nicotine, this substance has been reported to improve ADHD symptoms, but health issues associated with smoking seems to indicate that stopping the use of nicotine offers even more benefits for people with ADHD (van Amsterdam et al., 2018). Therefore, further *in vivo* research on polydrug reactions in ADHD is needed to fully understand these complex interactions and how it impacts on ADHD and its psychiatric comorbidities. Lastly, although this *in silico* modeling allowed us to get more insight into vLDX activity on BED and depression in patients with ADHD disease, which may be helpful to guide further research, the results obtained in this study must be validated or refuted with *in vitro* and/or *in vivo* data.

## 5. Conclusion

According to our *in silico* modeling study, vLDX’s targets were predicted to be related with high probability to proteins involved in BED as a comorbidity in patients with ADHD, for which LDX is currently indicated in the US adult population. In addition, vLDX was modeled to impact the pathophysiology of an ADHD comorbidity highly prevalent in adults and adolescents, namely, depression. Reasoned mechanisms were presented to explain our results other than neurotransmitter regulation, including effects on neuroplasticity, neuroinflammation, oxidative stress, and the HPA axis. These results could inform preclinical and even clinical future investigations treatments for patients with ADHD and psychiatric comorbidities.

## Data availability statement

The original contributions presented in the study are included in the article/Supplementary material, further inquiries can be directed to the corresponding author.

## Author contributions

JG-C, JQ, and CM designed the study. CS-V and MC performed the investigation and formal analyses. JG-C, JQ, JS, and TP-R contributed to data interpretation. JG-C and JQ drafted the first version of the manuscript. All authors critically revised all drafts of the manuscript for intellectual content.
