# Supplementary material for: In silico evaluation of the role of lisdexamfetamine on attention-deficit/hyperactivity disorder common psychiatric comorbidities: mechanistic insights on binge eating disorder and depression
Source: Front Neurosci. 2023 Jun 30;17:1118253. doi: 10.3389/fnins.2023.1118253 (PMC10347683; doi:10.3389/fnins.2023.1118253)
Supplement: Supplementary file 1 [file Data_Sheet_1.docx]

# *In silico* evaluation of the role of lisdexamfetamine on attention-deficit/hyperactivity disorder common psychiatric comorbidities: mechanistic insights on binge eating disorder and depression

***Supplementary Figures***

**José Ramón Gutiérrez Casares^1^٭, Cristina Segú-Vergés^2,3^, Juncal Sabate Chueca^4^, Tamara Pozo-Rubio^4^, Mireia Coma^2^, Carmen Montoto^4^**, **Javier Quintero^5^**

^1^ Unidad Ambulatoria de Psiquiatría y Salud Mental de la Infancia, Niñez y Adolescencia. Hospital Perpetuo Socorro. Badajoz, Spain

^2^ Anaxomics Biotech, Barcelona, Spain

^3^ Research Programme on Biomedical Informatics (GRIB), Departament de Ciències Experimentals i de la Salut, Universitat Pompeu Fabra, Barcelona, Spain

^4^ Medical Department. Takeda Farmacéutica España, Madrid, Spain

^5^ Servicio de Psiquiatría. Hospital Universitario Infanta Leonor. Universidad Complutense. Madrid, Spain

## Supplementary Figures

| 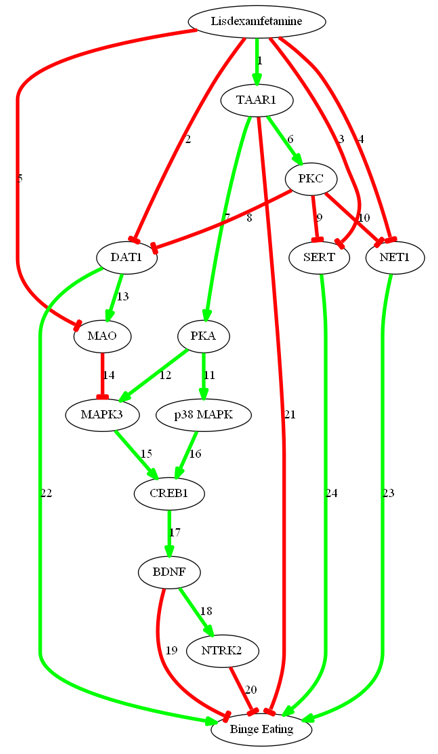 | |
| --- | --- |
| **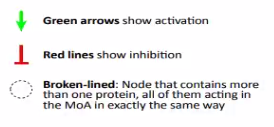** | **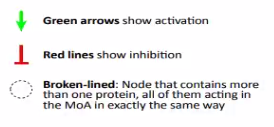** |
| **Figure A. Detailed predicted mechanism of action of virtual lisdexamfetamine over binge eating disorder in attention-deficit/hyperactivity disorder patients presenting this comorbidity.** Supplementary Table C in the S2 File contains the sources of information found in the scientific literature supporting the predicted mechanisms. | |

| 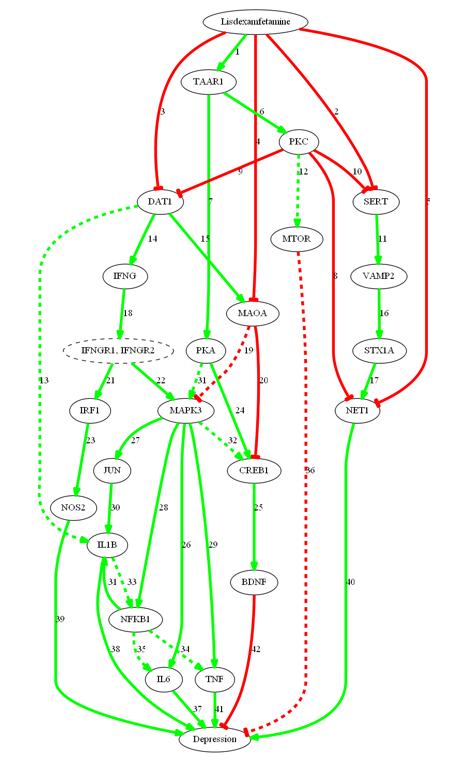  Detected  in  adults |
| --- |
| **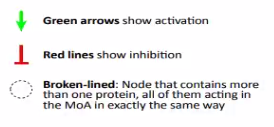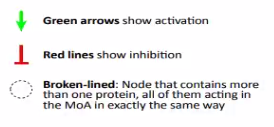**  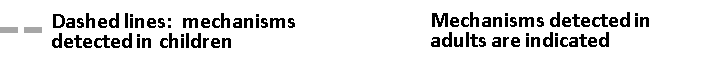 |
| **Figure B. Detailed predicted mechanism of action of virtual lisdexamfetamine over depression in attention-deficit/hyperactivity disorder patients presenting this comorbidity.** Supplementary Table D in the S2 File contains the sources of information found in the scientific literature supporting the predicted mechanisms. |
